# Rapid Recovery of CD3^+^CD8^+^ T Cells on Day 90 Predicts Superior Survival after Unmanipulated Haploidentical Blood and Marrow Transplantation

**DOI:** 10.1371/journal.pone.0156777

**Published:** 2016-06-08

**Authors:** Deng-Mei Tian, Yu Wang, Xiao-Hui Zhang, Kai-Yan Liu, Xiao-Jun Huang, Ying-Jun Chang

**Affiliations:** 1 Peking University People’s Hospital & Peking University Institute of Hematology, Beijing Key Laboratory of Hematopoietic Stem Cell Transplantation, No. 11 South Street of Xizhimen, Xicheng District, Beijing, China; 2 Peking-Tsinghua Center for Life Sciences, Beijing, China; 3 Collabrative Innovation Center of Hematology, Peking University, Beijing, China; 4 Department of Hematology, 309th Hospital, Chinese People’s Liberation Army, Beijing, China; Josep Carreras Leukaemia Research Institute, University of Barcelona, SPAIN

## Abstract

**Background:**

Rapid immune reconstitution after allogeneic hematopoietic stem cell transplantation (allo-HSCT) is significantly associated with lower infection, relapse and possibly secondary malignancy rates. The aim of this study was to investigate the role of peripheral lymphocyte subsets, especially CD3^+^CD8^+^ cytotoxic T cell recovery, in predicting transplant outcomes, including the overall survival (OS) and non-relapse mortality (NRM) rates after unmanipulated haploidentical blood and marrow transplantation (HBMT).

**Methods:**

Peripheral blood samples were obtained from 214 HBMT recipients with hematological malignancies. The peripheral lymphocyte subsets (CD3^+^ T cells, CD3^+^CD4^+^ helper T cells, CD3^+^CD8^+^ cytotoxic T cells, and CD19^+^ B cells) were analyzed by flow cytometry at days 30, 60, 90, 180, 270 and 360 after HBMT.

**Results:**

The CD3^+^CD8^+^ cytotoxic T cell recovery at day 90 (CD3^+^CD8^+^-90) was correlated with bacterial infection (*P* = 0.001), NRM (*P* = 0.001), leukemia-free survival (LFS, *P* = 0.005), and OS (*P* = 0.001) at a cutoff value of 375 cells/μL CD3^+^CD8^+^ T cells. The incidence of bacterial infection in patients with the CD3^+^CD8^+^-90 at ≥375 cells/μL was significantly lower than that of cases with the CD3^+^CD8^+^-90 at <375 cells/μL after HBMT (14.6% versus 41.6%, *P*<0.001). Multivariate analysis showed the rapid recovery of CD3^+^CD8^+^ T cells at day 90 after HBMT was strongly associated with a lower incidence of NRM (HR = 0.30; 95% CI: 0.15–0.60; *P* = 0.000) and superior LFS (HR = 0.51; 95% CI: 0.32–0.82; *P* = 0.005) and OS (HR = 0.38; 95% CI: 0.23–0.63; *P* = 0.000).

**Conclusion:**

The results suggest that the rapid recovery of CD3^+^CD8^+^ cytotoxic T cells at day 90 following HBMT could predict superior transplant outcomes.

## Introduction

Allogeneic hematopoietic stem cell transplantation (Allo-HSCT) is recognized as an effective treatment for patients with hematological malignancies. Successful immune reconstitution after allo-HSCT is significantly associated with lower infection, relapse and less secondary malignancy rates [1, 2]. This was attributed to repopulated lymphocytes that prevent infections and eradicate leukemia cells in the early phase after transplantation [3, 4]. In human leukocyte antigen (HLA)-identical sibling and/or matched unrelated donor (MUD) transplant settings, a lower absolute lymphocyte count on day 30 (ALC-30) predicted worse outcomes in patients receiving either T cell-depleted or unmanipulated grafts [5, 6]. Patients with myeloid leukemia and higher natural killer (NK) cell counts at day 30 had less relapses, a lower non-relapse mortality (NRM) and better survival [7]. Yet, in the pediatric HSCT cases, Koehl U et al. [8] reported that absolute CD3^+^CD8^+^ cytotoxic T cell counts above the 5th percentile of age-matched normal levels was independently associated with improved survival in the first year post-transplant. Additionally, in the umbilical cord blood transplantation (UCBT) setting, successful CD8^+^ T cell recovery was correlated with decreased leukemic relapse and better survival [9, 10]. However, there were some different views. Based on a cohort of 758 patients receiving BM allograft, Berger et al.[11] reported significantly improved survival and decreased NRM were due to rapid CD4^+^ helper T cell recovery rather than rapid NK-cell or CD8^+^-cell recovery. This result was consistent with a study by Kim et al. [12] showing that rapid CD4^+^ helper T cell recovery could predict overall survival (OS) and NRM.

Recently, we established an unmanipulated haploidentical blood and marrow transplantation (HBMT) protocol. The OS and leukemia-free survival (LFS) probabilities at 3 years in 756 patients undergoing unmanipulated HBMT were 67% and 63%, respectively [13]. Our preliminary study showed that patients who received HBMT experienced delayed early reconstitution of CD4^+^ T cells and dendritic cells that were accompanied by rapid CD3^+^CD8^+^ T cell and monocyte recovery [14]. However, it remains unclear whether the early recovery of T lymphocyte subsets was related with transplant outcomes after unmanipulated HBMTs. Therefore, we retrospectively analyzed T lymphocyte subset recovery in a large cohort of patients who received unmanipulated HBMT and assessed the impact of T lymphocyte subset recovery in transplant outcomes.

## Patients and Methods

### Patients

From January 2010 to December 2012, 214 consecutive patients underwent unmanipulated HBMTs at Peking University People’s Hospital, Peking University Institute of Hematology (Beijing, China). The patients were followed until the end of the study evaluation period in December 2014. Patients were excluded if they died or relapsed within 90 days after unmanipulated HBMT. Patients were included into the standard risk group if they were diagnosed with acute leukemia that was in first or second complete remission (CR) or chronic myelogenous leukemia (CML) in the chronic phase. Patients were classified into a high risk group if they were diagnosed with acute leukemia that was in more than the third CR, or if they had no remission, along with cytogenetic abnormalities, such as t(9;22) or t(4;11), CML in the accelerated or blast phase, or myelodysplastic syndrome-refractory anemia with excess blasts (MDS-RAEB) [15, 16].

### Ethics statement

This study protocol was approved by the Ethics Committee of Peking University People’s Hospital. Adult patients provided written informed consent prior to participation in this study. For the patients under the age of 18, written consent was provided by their parents or guardian.

### Transplantation

All of the patients were treated with myeloablative regimens, including a combination of 4 g/m^2^/d cytarabine on days -10 to -9, 3.2 mg/kg/d busulfan intravenously on days -8 to -6, 1.8 g/m^2^/d cyclophosphamide on days -5 and -4, 250 mg/kg simustine (MeCCNU) on day -3 and 2.5 mg/kg/d rabbit antihuman thymocyte immunoglobulin (ATG) (SangStat) intravenously on day -5 to -2 [15, 17].

The grafts included donor bone marrow and peripheral blood stem cells (PBSC). Donors received recombinant human granulocyte colony-stimulating factor (G-CSF) 5μg/kg daily for 5–6 d. On the 4th day, BM cells were harvested. BM and PBSCs were unmanipulated and infused fresh on the collection day. All patients received a combination of cyclosporine A, mycophenolate mofetil, and short-term methotrexate (MTX) for GVHD prophylaxis. MTX was administered intravenously at 15 mg/m^2^ on day +1 and 10 mg/m^2^ on days +3, +6 and +11 after transplantation. Cyclosporine was intravenously started at a dose of 2.5 mg/kg on day -9 and switched to oral medication until the patients were able to tolerate it. The cyclosporine dose was adjusted to maintain blood levels between 150 ng/mL and 250 ng/mL. Oral mycophenolate mofetil (0.5 g every 12 hours) was started at day -9 and day +30, then tapered and discontinued until day +60. Exposure to systemic steroids was defined as therapy with at least 0.5 mg/kg methylprednisolone or prednisone, or dexamethasone for 7 or more consecutive days within 90 days after transplantation. All of the recipients were subcutaneously injected with G-CSF (5 μg/kg daily) from day +6 after transplantation until their neutrophil counts were over 0.5×10^9^ cells/L for 3 consecutive days. Day +30, +60, +90 donor chimerism and engraftment were assessed from BM aspiration and/or peripheral blood. Polymerase chain reaction (PCR)-DNA fingerprinting and HLA DNA typing were performed to determine donor chimerism [18].

### Donor 1ymphocyte infusion (DLI)

DLI protocol was implemented as published previously [19]. Indications for DLI included: (1) patients were classified into a high risk group as mentioned above; (2) hematological leukemia relapse; Patients received chemotherapy, followed by DLI; (3) molecular tests provided evidence of persistent leukemia or a recurrence in patients without GVHD.

### Outcome definitions

Neutrophil engraftment was defined as when the absolute neutrophil count exceeded 0.5×10^9^/L for 3 consecutive days after transplantation. The time of engraftment was counted as the first of these 3 consecutive days. Similarly, platelet engraftment was considered as the time when a blood platelet count after transplantation exceeded 20×10^9^/L without transfusion support for 7 consecutive days. Acute and chronic GVHD were assessed and graded based on the standard criteria. OS was defined as the time from the beginning of transplantation to death from any cause. LFS was defined as the time elapsed from the beginning of transplantation to a relapse. NRM was defined as the time from transplantation until death from all causes other than those directly related to a hematologic malignant disease itself. A definite infection was diagnosed when a positive culture or PCR for a pathogen was detected in association with clinical symptoms and signs. Additionally, infections were presumed based on the combination of clinical presentation, imaging findings and response to treatment with antibiotics (for example, all microbiologically undocumented pneumonias that were cured by empirical antibiotics treatment were considered as infections).

### Immunophenotyping

Peripheral blood samples were collected from recipients at days 30, 60, 90, 180, 270 and 360 after HBMT. The samples were stained without further separation to minimize selective loss shortly after collection. The combinations of the directly conjugated monoclonal antibodies CD3-FITC, CD4-PE, CD8-APC, CD19-Per-CP (BD Biosciences, Mountain View, CA, USA), and their isotype-matched antibodies were used to analyze the immunophenotype of T lymphocyte subsets. Flow cytometry was performed using a BD FACSSort machine (Becton Dickinson Biosciences, San Jose, CA, USA).The data were analyzed using CellQuest software (BD Biosciences).

### Endpoint and Statistical analysis

The primary endpoint of the study was to evaluate whether the CD3^+^CD8^+^ T cell count on day 90 after transplantation (CD3^+^CD8^+^-90) had an impact on the OS. To avoid the influence of relapse on the CD3^+^CD8^+^ T cells, we performed a landmark analysis and excluded relapse or death within 90 days after HBMT. A receiver operating characteristic (ROC) curve was used to find the lymphocyte subset cut-off value, as was previously reported [20, 21]. Patients were assigned to groups with “high” or “low” cell count (CD3^+^CD8^+^-90 ≥375 cells/μL or CD3^+^CD8^+^-90 <375 cells/μL, respectively). The Kaplan-Meier estimate was used to estimate the time-to-event distributions of the OS and LFS. Log-rank test was used to assess survival. Cumulative incidences for NRM and relapse were estimated and competing events were defined as relapse for NRM and non-relapse death for relapse, respectively. Gray test was used to analyze cumulative incidence. Univariate and multivariate analyses in Cox proportional hazard models were used to assess proportional hazards assumptions and for testing interaction terms with covariates. The variables included in the multivariate analysis were as follows: age, sex, donor-recipient sex match, disease status, ABO match, HLA match, CD34^+^ cell dose, CD3^+^CD8^+^ T cell count on day 90 post-transplantation, donor lymphocyte infusion (DLI), and occurrence of acute GVHD (aGVHD) (grade II-IV) and any grade of chronic GVHD (cGVHD). The occurrence of DLI and aGVHD and cGVHD were included as time dependent variables in multivariable analysis. The Mann-Whitney U (for continuous variables) and chi-square statistics (for categorical variables) tests were used to compare differences between the groups. Repeated measurement of the general linear model was used for the series recovery for each lymphocyte subset in patients after transplantation until day 360, according to the cutoff value of the lymphocyte subset. A linear regression analysis was used to analyze lymphocyte subset correlations. *P*-values <0.05 were regarded as significant. All of the statistical analyses were performed with SPSS Version 19.0 software. R software was used to calculate the cumulative incidence (version 2.15.2, the CRAN project).

## Results

### Patient Characteristic

The baseline characteristics of the patients are shown in Table 1. A total of 214 patients (71.5% males and 28.5% females) were transplanted. The median age was 23.5 years (range, 3 to 55). The median follow-up time was 29.4 months (range, 3.3 to 59.6). The main causes of death were relapse of the underlying malignancy (n = 25) or NRM, including severe infections (n = 24) and severe GVHD (n = 11). In the present study, 41 patients received a DLI for either a therapeutic DLI (n = 23) or a prophylactic DLI (n = 18) based on our protocol, which was published previously [19]. None of patients in this study received a DLI within 90 days after HBMT, which excluded an effect of DLI on lymphocyte subsets recovery. The median time from transplant to DLI was 276 days (range, 96–901). The median dose of infused mononuclear cells, CD3^+^ cells, CD4^+^ cells, and CD8^+^ cells were 1.11 (range, 1.00 to 3.14) × 10^8^/kg, 0.34 (range, 0.15–1.62) × 10^8^/kg, 0.17 (range,0.01–0.92) × 10^8^/kg, and 0.18 (range, 0.01–0.52) × 10^8^/kg, respectively.

**Table 1:** Patient characteristics.

Variable	Total Patient (n = 214)	CD3^+^CD8^+^-90 ≥375 cells/μL(n = 137)	CD3^+^CD8^+^-90 <375 cells/μL(n = 77)	*P*
Age of recipients at allo-HSCT, y*				
Median (range)	23.5(3–55)	22(3–55)	26(5–54)	0.348
**Sex, no.(%)**^**&**^				
Male	153(71.5)	95(69.3)	58(75.3)	0.352
Female	61(28.5)	42(30.7)	19(24.7)	
**Diagnosis, no.(%)**^**&**^				
ALL	76(35.5)	54(39.4)	22(28.6)	0.100
AML	93(43.5)	53(38.7)	40(51.9)	
CML	20(9.3)	11(8.0)	9(11.7)	
MDS	14(6.5)	9(6.6)	5(6.5)	
NHL	9(4.2)	8(5.8)	1(1.3)	
AHL	2(0.9)	2(1.5)	0(0.0)	
**Disease status at transplantation, no. (%)**^**&**^				
Standard/high risk	183(85.5)/31(14.5)	115(83.9)/22(16.1)	68(88.3)/9(11.7)	0.383
Time from diagnosis to transplantation, d*				
Median (range)	180(30–3600)	180 (30–3600)	180 (60–1080)	0.659
Age of donors at allo-HSCT, y*				
Median (range)	41(14–64)	41(15–64)	41(14–63)	0.860
**Donor-recipient sex match, no. (%)**^**&**^				
male—male	101(47.2)	63(46.0)	38(49.4)	0.796
male—female	38(17.8)	25(18.2)	13(16.9)	
female—male	54(25.2)	33(24.1)	21(27.3)	
female—female	21(9.8)	16(11.7)	5(6.5)	
**Donor-recipient HLA match, no. (%)**^**&**^				
One locus mismatch	15(7.0)	9(6.6)	6(7.8)	0.632
Two locus mismatch	64(29.9)	44(32.1)	20(26.0)	
Three locus mismatch	135(63.1)	84(61.3)	51(66.2)	
**Donor-recipient ABO match, no. (%)**^**&**^				
Matched	114(53.3)	68(49.6)	46(59.7)	0.640
Major mismatched	56(26.2)	40(29.2)	16(20.8)	
Minor mismatched	44(20.6)	29(21.2)	15(19.5)	
**Donor-recipient relationship, no. (%)**^**&**^				
Father-child	97(45.3)	66(48.2)	31(40.3)	0.237
Mother-child	44(20.6)	31(22.6)	13(16.9)	
Sibling-sibling	52(24.3)	28(20.4)	24(31.2)	
Child-parent	21(9.8)	12(8.8)	9(11.7)	
**Infused nuclear cells in graft (×10**^**8**^/kg, range)*	8.03(2.2–18.21)	8.02(3.4–18.21)	8.06(2.2–13.53)	0.634
**Infused CD3**^**+**^ **cells in graft (×10**^**8**^/kg, range)*	1.6(0.12–8.69)	1.57(0.12–8.69)	1.75(0.79–3.7)	0.021
**Infused CD34**^**+**^ **cells in graft (×10**^**6**^/kg, range)*	2.58(0.3–20.54)	2.65(0.3–20.51)	2.55(0.46–8.32)	0.589
**Steroid therapy within 90 days after transplant**				
Yes	137(64.0)	87(63.5)	50(64.9)	0.834
No	77(36.0)	50(36.5)	27(35.1)	
Interval from HSCT to intervention of DLI, d*				
Median (range)	276(96–901)	330(124–850)	275(115–901)	0.282

AML, acute myeloid leukemia; ALL, acute lymphoblastic leukemia; CML, chronic myeloid leukemia; MDS, myelodysplastic syndrome; AHL, acute myeloid and lymphoblastic leukemia; NHL, non-Hodgkin lymphoma; allo-HSCT, allogeneic hematopoietic stem cell transplant; d, days; y, years.

*Mann-Whitney U test was used to calculate *P* values.

^&^Chi-square test was used to calculate *P* values.

Disease status at transplantation: Patients were included into standard risk group when being diagnosed acute leukemia in first or second CR or CML in the chronic phase. Patients were classified as high risk group when being diagnosed acute leukemia in more than the third CR or no remission, with cytogenetic abnormalities such as t(9;22) or t(4;11), CML in the accelerated or blast phase, myelodysplastic syndrome-refractory anemia with excess blasts (MDS-RAEB).

### Lymphocyte subsets recovery after unmanipulated HBMT

**Fig 1:**
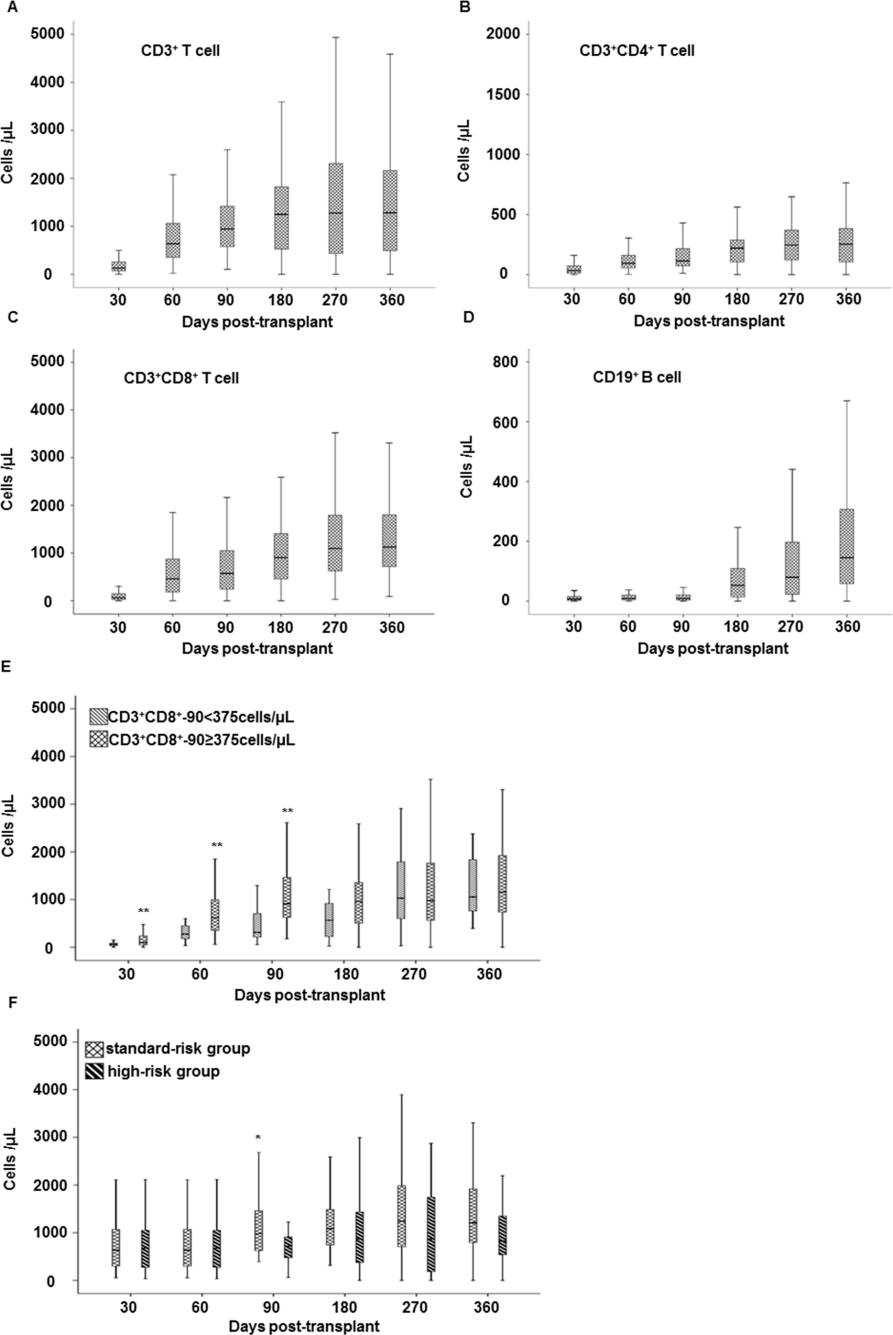
Lymphocyte subset recovery after unmanipulated HBMT. The median of each lymphocyte subset gradually rose on days 30, 60, 90, 180, 270, and 360 post-transplant, and included: (**A)** CD3^+^ T cells; (**B)** CD3^+^CD4^+^ helper T cells; (**C)** CD3^+^CD8^+^ cytotoxic T cells; and (**D)** CD19^+^ B cells. (**E)** Differences in the CD3^+^CD8^+^-90 T cell recovery for patients with CD3^+^CD8^+^-90 ≥375 cells/μL and those with CD3^+^CD8^+^-90 <375 cells/μL and (**F)** differences in the CD3^+^CD8^+^-90 T cell recovery for standard-risk and high-risk patients are shown. *P<0.05, **P<0.01.

The immune recovery pattern was shown for each lymphocyte subset. In general, the median CD3^+^ T cells, CD3^+^CD4^+^ T cells, CD3^+^CD8^+^ T cells and CD19^+^ B cell level gradually rose on days 30, 60, 90, 180, 270, and 360 (Fig 1). The CD3^+^CD4^+^ T-cell and CD19^+^ B-cell recovery was significantly delayed compared with the CD3^+^CD8^+^ T cells (Fig 1B–1D). The CD3^+^CD8^+^ T cell counts dropped after conditioning and were significantly reduced on day 30 after transplantation. Thereafter, the absolute CD3^+^CD8^+^ T cell counts were dramatically expanded (Fig 1C). Moreover, the CD4^+^/CD8^+^ T-cell ratio was continuously inverted during the first year after transplantation.

According to previous literature [20, 21], the cutoff value of every lymphocyte subset was calculated for the clinical OS prediction at different time points after transplantation through a ROC curve. Except for the CD3^+^CD8^+^ T cell counts on day 90 (CD3^+^CD8^+^-90), there was no significant difference in the other subsets. The cutoff value was identified by the CD3^+^CD8^+^-90 level at ≥375 cells/μL or <375 cells/μL. The patients were divided into two groups: “high” (CD3^+^CD8^+^-90 cells ≥375 cells/μL) and “low” (CD3^+^CD8^+^-90 cells <375cells/μL). The patient characteristics between the two groups were not significantly different (Table 1). 64% of the patients recovered CD3^+^CD8^+^ T-cell counts above 375 cells/μL on day 90 after transplantation. At days 30, 60, and 90, the CD3^+^CD8^+^ T cell recovery in the patients in the high cells group was significantly faster than in those in the low cells group (Fig 1E). Moreover, the CD3^+^CD8^+^ T cell recovery at day 90 after transplantation in the standard-risk patients was faster than that in the high-risk group (Fig 1F).

**Fig 2:**
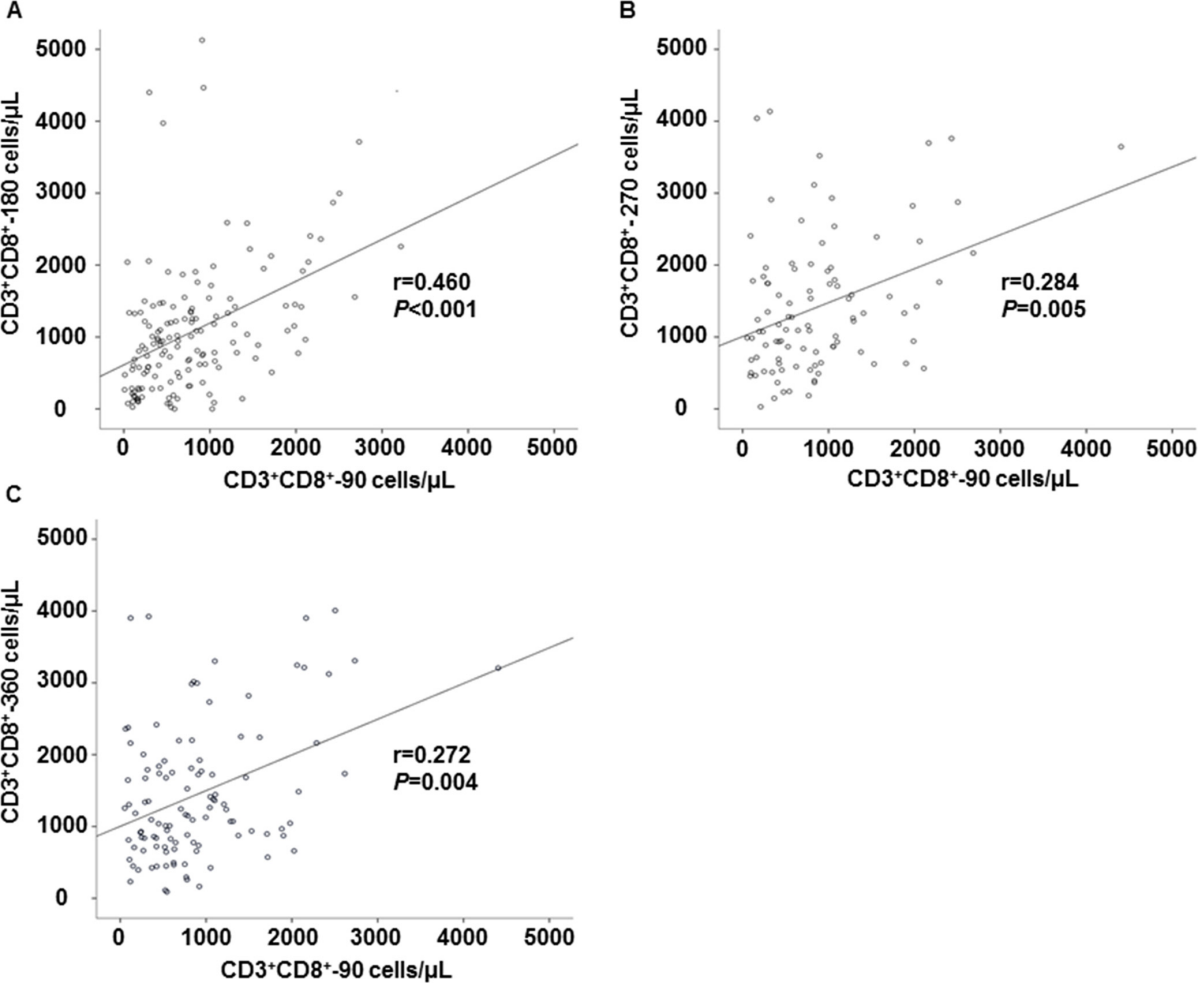
The impact of the CD3^+^CD8^+^ T cell recovery after unmanipulated HBMT on subsequent immune recovery, until day 360. **(A)** Correlation between the CD3^+^CD8^+^ T-cell recovery at days 90 and 180; (**B)** Correlation between the CD3^+^CD8^+^ T-cell recovery at days 90 and 270; (**C)** Correlation between the CD3^+^CD8^+^ T-cell recovery at days 90 and 360. The r-value represents the Spearman’s rank correlation coefficient; *P* value represents the significance of the correlation.

Regarding the impact of the CD3^+^CD8^+^-90 T-cell counts on the subsequent immune recovery, we observed that the absolute counts for each lymphocyte subset, including the CD3^+^ T-cells, CD3^+^CD4^+^ T cells, CD3^+^CD8^+^ T cells and CD19^+^ B cells at days 180, 270 and 360, rose faster in the patients in the high cell group compared with those in the low cell group (Table 2). The linear regression analysis showed that a rapid recovery of CD3^+^CD8^+^ T cell on day 90 was strongly correlated with a subsequent CD3^+^CD8^+^ T-cell reconstitution from 180 to 360 days after transplantation (Fig 2). Meanwhile, repeated measurements of the general linear model were also used to further confirm the difference in the sequential recovery pattern for every subset. The CD3^+^CD8^+^ T-cell recovery on day 90 had a significant impact on their subsequent reconstitution, including on days 180 (*P*<0.001), 270 (*P* = 0.005), and 360 (*P* = 0.004). However, this effect was not observed in the other lymphocyte subsets.

**Table 2:** CD3^+^CD8^+^ T-cell counts at day 90 and its impact on subsequent immune recovery until day 360.

Mean ± S.E. (cells/μL)	90d	180d	270d	360d
**No. examined**				
high cell group	137	105	93	83
low cell group	77	62	50	47
**CD3**^**+**^ T cells*				
high cell group	1432±73	1608±94	1867±128	1252±241
low cell group	637±92	1431±200	1765±265	1157±127
*P* value	<0.001	0.034	0.428	0.468
**CD3**^**+**^**CD4**^**+**^ T cells*				
high cell group	203±15	249±18	328±27	380±31
low cell group	88±10	222±24	273±49	341±57
*P* value	<0.001	0.504	0.096	0.206
**CD3**^**+**^**CD8**^**+**^ T cells*				
high cell group	1111±58	2344±148	1441±106	1470±104
low cell group	178±12	773±116	1368±216	1375±182
*P* value	<0.001	<0.001	0.481	0.565
**CD19**^**+**^ B cells*				
high cell group	38±5	98±10	168±19	250±23
low cell group	26±7	99±18	84±23	143±23
*P* value	<0.001	0.542	0.003	0.028

S.E., standard error; No., patient number. high cell group: patients with CD3^+^CD8^+^-90≥375cells/μL. low cell group: patients with CD3^+^CD8^+^-90<375 cells/μL. *Mann-Whitney U test was used to calculate *P* values.

### Effects of CD3^+^CD8^+^-90 T cell counts on transplant outcomes

#### GVHD

Amongst the 214 patients, 52 patients (24.3%) developed grade II to IV acute GVHD (aGVHD), a total of 214 people survived more than 100 days, 93 of whom (43.5%) developed chronic GVHD (cGVHD) (limited, 54/214, 25.2%; extensive, 39/214, 18.2%). After a median follow-up of 29.4 months (range, 3.3–59.6months), the cumulative incidence of total cGVHD was 50.8% (95% CI, 0.45%-57%) in the CD3^+^CD8^+^-90<375 cells/μL group, comparable to the rates in the CD3^+^CD8^+^-90≥375 cells/μL group (55.9%; 95% CI, 52%-59%; *P* = 0.879). No significant differences were observed regarding the aGVHD and cGVHD incidence rates between the two groups after unmanipulated HBMT (Table 3). After DLI interventions, the cumulative incidence of grade II to IV aGVHD was no significant difference between the two groups (43% vs. 0.38%, *P* = 0.361). Also, there were no significant differences in the incidences of total cGVHD between the two groups (39.3% vs 41.2%, *P* = 0.431).

**Table 3:** Clinical outcomes of patients after transplantation based on CD3^+^CD8^+^-90 T cell counts.

Variable	CD3^+^CD8^+^-90≥375 cells/μL(n = 137)	CD3^+^CD8^+^-90<375 cells/μL (n = 77)	*P*
**Acute GVHD, no. (%)&**			
None	80(58.4)	43(55.8)	0.201
Grade I	27(19.7)	12(15.6)	
Grade II	27(19.7)	15(19.5)	
Grade III	3(2.2)	6(7.8)	
Grade IV	0(0)	1(1.3)	
**Chronic GVHD, no. (%)&**			
None	78(56.9)	43(55.8)	0.606
Limited	32(23.4)	22(28.6)	
Extensive	27(19.7)	12(15.6)	
**Infectious events, no. (%)&**			
Bacteria	20(14.6)	32(41.6)	<0.001
Fungus	18(23.4)	19(13.9)	0.078
CMV	84(61.3)	55(71.4)	0.137
**NRM, no. (%)&**	15(10.9)	22(28.6)	<0.001
Infection	4(2.9)	14(18.2)	
GVHD + Infection	4(2.9)	2(2.6)	
GVHD	2(1.5)	3(3.9)	
others	1(0.7)	1(1.3)	
**Relapse, no. (%)&**	21(15.3)	14(18.1)	0.282
**LFS, no. (%)&**	101(73.7)	41(53.2)	0.001
**Length of follow-up post-transplant, mo#**	29.4 (3.3–59.6)		

NRM, non-relapse mortality; LFS, Leukemia-free survival; mo, months.

#Median (range).

&Chi-square test was used to calculate P values.

#### NRM

**Fig 3:**
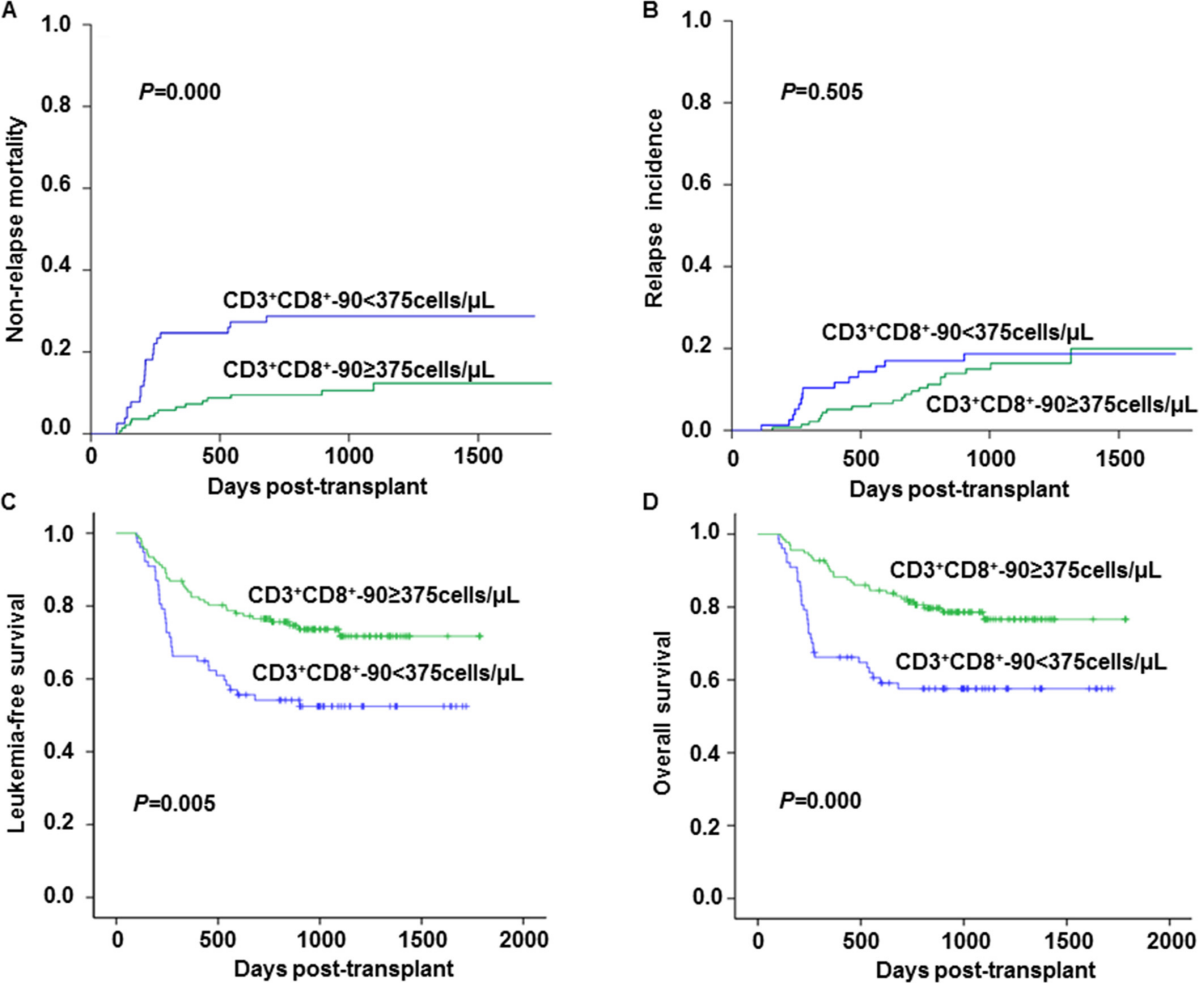
The impact of the CD3^+^CD8^+^-90 T cell recovery after unmanipulated HBMT on transplantation outcome. **(A)** Cumulative incidence of non-relapse mortality; (**B)** cumulative incidence of relapse; **(C)** leukemia-free survival; **(D)** overall survival rates, according to CD3^+^CD8^+^-90 T cell counts at days 90.

A total of 37 amongst 214 (17.3%) patients died from non-relapse causes after unmanipulated HBMT. The main causes of death included GVHD-related death (11 cases), bacterial or fungal or viral fatal infection (24 cases), post-transplant lymphoproliferative disorder (1 case), and pulmonary hemorrhage (1 case). The univariate analysis showed that factors associated with a low NRM included CD3^+^CD8^+^-90 T cell counts ≥375 cells/μL (*P* = 0.001) (Table 4). In the multivariate analysis, the high CD3^+^CD8^+^-90 T cell counts were independently associated with a decreased NRM risk (HR: 0.30; 95% CI: 0.15–0.60; *P* = 0.000) (Fig 3A, Table 5). Additionally, we also investigated association of the CD3^+^CD8^+^-90 T cell counts and infection. The patients with a rapid recovery of CD3^+^CD8^+^-90 T cells showed a much lower incidence of bacterial infection compared to those with a delayed reconstitution (14.6% versus 41.6%, *P*<0.001) (Table 3). Details on microbiological species of infections were shown in the S1 Table. Among them, gram-negative infection was the most frequent. Pneumonias were also the most common infections, which accounted for 60% of documented bacterial infections and were the main cause of death. However, the incidence of the fungal infection and cytomegalovirus (CMV) reactivation were not significantly different in patients with CD3^+^CD8^+^-90 ≥375 cells/μL compared with those with CD3^+^CD8^+^-90 <375 cells/μL (Table 3). We found the CD3^+^CD8^+^-90 ≥375 cells/μL was independently associated with a reduced risk of bacterial infection (HR: 0.24; 95% CI: 0.10–0.56; *P* = 0.001) (Table 5).

**Table 4:** Univariate Analysis of factors associated with infection, relapse, NRM, LFS and OS.

Variable	Bacterial infection		Relapse		NRM		LFS		OS	
	HR (95%CI)	*P*	HR (95%CI)	*P*	HR (95%CI)	*P*	HR (95%CI)	*P*	HR (95%CI)	*P*
**Age of recipients**	1.00(0.96–1.04)	0.888	1.02 (0.97–1.07)	0.403	0.97(0.91–1.03)	0.313	1.01(0.97–1.04)	0.786	1.00(0.96–1.04)	0.888
**Gender of recipients**	0.00(0.00–1.75)	0.838	0.01(0.00–1.26)	0.875	0.00(0.00–5.05)	0.904	0.00(0.00–3.86)	0.808	0.00(0.00–1.75)	0.838
**Diagnosis**	0.82(0.61–1.11)	0.203	0.62 (0.40–0.96)	0.033	0.83(0.54–1.28)	0.398	0.71(0.53–0.95)	0.021	0.82(0.61–1.11)	0.203
**Disease status**	1.27(0.58–2.78)	0.555	2.802(0.91–8.63)	0.073	1.00(0.38–2.65)	0.994	1.90(0.90–3.99)	0.092	1.27(0.58–2.78)	0.555
**Age of donors**	0.98(0.95–1.02)	0.324	0.96(0.92–0.99)	0.012	1.01(0.95–1.08)	0.723	0.98(0.95–1.01)	0.244	0.98(0.95–1.02)	0.324
**Time from diagnosis to transplantation**	1.00(0.99–1.00)	0.621	1.00(1.00–1.01)	0.388	1.00(0.99–1.00)	0.749	1.00(0.99–1.00)	0.622	1.00(0.99–1.00)	0.621
**Donor-recipient sex match**	1.25(0.62–2.51)	0.537	1.06 (0.47–2.41)	0.884	1.20(0.44–3.27)	0.717	0.93(0.52–1.67)	0.810	1.25(0.62–2.51)	0.537
**Donor-recipient HLA match**	0.92(0.62–1.37)	0.677	0.84(0.47–1.53)	0.575	1.10(0.67–1.80)	0.718	1.05(0.73–1.51)	0.783	0.92(0.62–1.37)	0.677
**Donor-recipient ABO match**	0.87(0.63–1.19)	0.371	0.83 (0.54–1.30)	0.418	0.92(0.61–1.37)	0.666	0.90(0.67–1.21)	0.476	0.87(0.63–1.19)	0.371
**Donor-recipient relationship**	0.94(0.51–1.74)	0.844	0.64(0.30–1.34)	0.235	1.46(0.53–4.07)	0.466	0.93(0.53–1.63)	0.795	0.94(0.51–1.74)	0.844
**chronic GVHD**	3.53(2.10–5.92)	0.000	0.41(0.19–0.87)	0.020	13.9(6.27–30.7)	0.000	2.09(1.33–3.29)	0.001	3.53(2.10–5.92)	0.000
**CD3**^**+**^**CD8**^**+**^**-90 T cell counts**	0.41(0.24–0.70)	0.001	0.57 (0.27–1.21)	0.141	0.28(0.13–0.61)	0.001	0.48(0.29–0.80)	0.005	0.41(0.24–0.70)	0.001
**Infused CD3**^**+**^ **cell dose**	0.87(0.59–1.29)	0.480	0.66(0.39–1.11)	0.118	0.95(0.54–1.67)	0.862	0.73(0.51–1.05)	0.092	0.87(0.59–1.29)	0.480
**CD34**^**+**^ **cell dose**	0.97(0.81–1.17)	0.754	1.01(0.80–1.27)	0.952	1.02(0.80–1.29)	0.896	1.02(0.86–1.20)	0.860	0.97(0.81–1.17)	0.754
**DLI**	1.97(1.40–2.78)	0.000	0.26(0.15–0.44)	0.000	1.56(0.98–2.47)	0.062	0.40(0.29–0.56)	0.000	0.51(0.36–0.71)	0.000

OS, overall survival; NRM, non-relapse mortality; LFS, Leukemia-free survival; HLA, human leukocyte antigen; DLI, donor lymphocyte infusion; HR, hazard ratio; CI, confidence interval.

**Table 5:** Multivariate analysis of factors affecting OS, LFS, relapse, NRM and bacterial infection in patients after transplantation.

Outcome	HR	95% CI	*P*
**OS**			
CD3^+^CD8^+^-90 T cell counts (**≥375 cells/**μ**L)**	0.38	0.23–0.63	0.000
DLI (yes)	0.52	0.38–0.72	0.000
chronic GVHD	3.49	2.15–5.66	0.000
**LFS**			
CD3^+^CD8^+^-90 T cell counts (**≥375 cells/**μ**L)**	0.51	0.32–0.82	0.005
DLI (yes)	0.42	0.24–0.31	0.000
**Relapse**			
DLI (yes)	0.25	0.16–0.41	0.000
**NRM**			
CD3^+^CD8^+^-90 T cell counts (**≥375 cells/**μ**L)**	0.30	0.15–0.60	0.000
**Bacterial infection**			
CD3^+^CD8^+^-90 T cell counts (**≥375 cells/**μ**L)**	0.24	0.10–0.56	0.001

OS, overall survival; NRM, non-relapse mortality; LFS, Leukemia-free survival; DLI, donor lymphocyte infusion; HR, hazard ratio; CI, confidence interval.

#### Relapse

There were no significant relapse differences in the patients with CD3^+^CD8^+^-90 <375 cells/μL compared with those with CD3^+^CD8^+^-90≥375 cells/μL (18.1% versus 15.3%, *P* = 0.282) (Table 3, Fig 3B). However, the LFS of the former was significantly inferior to that of the latter (53.2% versus 73.7%, *P* = 0.001) (Table 3, Fig 3C). Additionally, the disease relapse incidence was significantly increased in the high-risk patients (HR: 2.80; 95% CI: 0.91–8.63; *P* = 0.073) (Table 4). In our study, 15 of the 23 relapsed patients who underwent therapeutic DLI achieved a complete recovery, and 10 of 15 patients were alive and did not suffer from leukemia until the end of the follow-up period. 5 cases relapsed in the 18 patients that received prophylactic DLI. In addtion, a chimerism analysis showed the BM samples of all relapsed patients were mixed donor chimerism, and patients achieving a complete remission after a therapeutic DLI showed full donor chimerism. The multivariate analysis indicated that DLI was significantly correlated with a lower disease relapse risk (HR: 0.25; 95% CI: 0.16–0.41; *P* = 0.000) (Table 5).

#### OS

Amongst the 214 patients, 153 were alive after their transplantations. The patients with CD3^+^CD8^+^-90≥375 cells/μL had an improved median OS compared with those with CD3^+^CD8^+^-90<375 cells/μL (median OS, 30.7 months versus 21.2 months, *P* = 0.000) (Fig 3D). Chronic GVHD, CD3^+^CD8^+^-90 T cell counts and DLI were significantly associated with OS in the univariate analyses. Other variables were not associated with OS, including patient age, sex, time from diagnosis to transplantation, and CD34^+^ cell dose (Table 4). A multivariate Cox regression analysis showed that higher CD3^+^CD8^+^-90 T cell counts (≥375 cells/μL) (HR: 0.38; 95% CI: 0.23–0.63; *P* = 0.000) and DLI (HR: 0.52; 95% CI: 0.38–0.72; *P* = 0.000) were significantly associated with improved OS (Table 5).

#### Factors associated with CD3+CD8+ T-cell recovery at day 90 post-transplantation

As shown above, a higher CD3^+^CD8^+^-90 T-cell count could predict better survival. Here, we analyzed potential factors that could impact the CD3^+^CD8^+^ T-cell recovery. Infused CD3^+^ T cell dose (*P* = 0.031), the time from diagnosis to transplantation (*P* = 0.029), and bacterial infection (*P* = 0.000) were significantly associated with CD3^+^CD8^+^ T cell recovery in the univariate analyses. However, the infused CD3^+^ cell dose was the only factor that correlated with the CD3^+^CD8^+^ T cells recovery in the multivariate analysis (HR: 0.67; 95% CI: 0.50–0.90; *P* = 0.008).

## Discussion

The current study assessed the influence of lymphocyte subset recovery on transplantation outcome in a large cohort of patients. We found that patients with the CD3^+^CD8^+^-90≥375 cells/μL experienced lower NRM incidence and superior LFS and OS rates. These results added a new variable in predicting transplantation outcomes for patients who undergo unmanipulated HBMT, suggesting that early CD3^+^CD8^+^ cytotoxic T-cell recovery might have predictive significance not only in UCBT [9, 10], HLA-identical sibling and MUD-HSCT [5, 6] but also in haploidentical HSCT.

Studies from mouse models and in humans have confirmed the effects of CD8^+^ T cells in protecting recipients against infections and leukemic relapses [22, 23]. Our study demonstrated that there was a lower incidence of bacterial infection among the patients with a higher CD3^+^CD8^+^-90 T cell count compared with those with a lower count. These might be attributed to the bifunctional properties of CD8^+^ T cells, including their interferon-γ production and cytolytic activity [24]. Luo et al. [25] found that patients with self-resolving or treated infections had higher central memory cytomegalovirus-specific CD8^+^ T lymphocyte levels compared with those with undetected infections. These central memory CD8^+^ T cells might have a stronger antitumor and/or antivirus immunity compared with the observed effector memory T cells [25, 26]. Moreover, studies demonstrated that early recovery of T lymphocytes, especially CD8^+^ cells, are believed to occur because donor-derived T cells undergo rapid expansion by alloantigen and high concentration cytokine stimulation. This substantially repopulates the total T cell pool, which accordingly was shown to contribute to the production and increase of CD8^+^ T cells early post-transplantation [27, 28]. In addition, Oghumu et al. demonstrated that CXCR3^+^ subpopulations of CD8^+^ T cells could potentially provide enhanced immune responses against bacterial infection [29, 30]. Maybe this needs to be further explored in our transplantation model. On the other hand, previous studies showed that CD4^+^ helper T cells had an important effect on transplant outcomes. Kim et al. [12] reported that patients with a CD4^+^ T-cell recovery above 2×10^8^ cells/L at 3 months after allogeneic HSCT had a significantly better clinical outcome. This report was consistent with a study by Berger et al. [11]. However, in our transplantation model, we did not find an association between CD4^+^ T-cell recovery and clinical outcomes. This difference may involve different allografts, conditioning regimens, and HLA matching degrees between our transplantation model and others. However, we cannot exclude the role of CD4^+^ T-cell recovery in preventing infections.

Relapse has been a major reason for transplant failure after allo-HSCT. Previous studies showed that patients with a high ALC early after transplantation had improved survival, suggesting that a rapid lymphocyte recovery might benefit the occurrence of graft-versus-leukemia (GVL) effects [4]. However, in the present study, we failed to demonstrate an association between high CD3^+^CD8^+^-90 T cell levels and a low incidence of relapse. However, patients with the higher CD3^+^CD8^+^-90 T cell count had better LFS. Possible explanations for this include: (1) prophylactic or therapeutic DLI may contribute to decreased relapse. Yan et al. [19] showed that our DLI protocol didn’t increase DLI-associated severe GVHD and kept the benefit of GVL effects, and (2) other immune cells, such as CD4^+^ helper T-cell counts and natural killer cells, may be effective cells in eradicating leukemia cells in spite of low counts after transplantation [31, 32]. However, a role for the reconstituted CD8^+^ T cells after unmanipulated HBMT in eliminating leukemia cells could not be excluded.

The association of a high CD3^+^CD8^+^-90 T-cell count with better survival prompted us to assess factors that are involved in immune recovery. We found that the infused CD3^+^ T cell dose significantly affected the degree of CD3^+^CD8^+^ T-cell recovery. Dong et al. [33] reported that patients infused with a relatively high dose of CD3^+^ cells had less transplant-related mortality (TRM) and a more intensive GVL effect, without increased GVHD. Combined with our previous result that the CD3^+^ cell dose in graft was associated with ALC-30 reconstitution [34], it is reasonable that infused donor-derived CD3^+^ T cells might affect T cell reconstitution at different time points after unmanipulated HBMT. Studies demonstrated that T cell recovery involves two different mechanisms: (1) homeostatic expansion of transferred donor T cells (thymus-independent) and (2) thymic selection and generation of naive thymic T cells [11, 26]. Our results suggest that increasing the CD3^+^ T-cell dose may not only contribute to early T-cell reconstitution but also to late reconstitution.

In a previous study, Chang et al. [32] showed that patients with more CD56^bright^ NK cells at days 14 post unmanipulated HMBT had a higher survival rate. Furthermore, patients with a higher ALC-30 after unmanipulated HBMT were associated with better survival [35]. The results found in this study and together with our previous findings suggest that patients receiving unmanipulated HBMT can be monitored continuously at different time points using different parameters to identify ones who are in the high-risk of infections and/or relapse.

Our data are limited by the characteristics inherent to single center, retrospective analyses. Additionally, the functional analysis of T cell subsets, including CD3^+^CD8^+^ cytotoxic T cells, were not included. Therefore, a prospective, multicenter study is warranted to confirm the results observed in this study.

In summary, this study showed that a high number of CD3^+^CD8^+^ T cells at day 90 after unmanipulated HBMT was an independent variable that can predict lower NRM and superior survival. These results together with the data reported by others suggest that reconstituted CD3^+^CD8^+^ cytotoxic T cells can be an indicative and reasonably monitored variable to recognize the need of immunotherapeutic intervention before the occurrence of relapse and/or life-threatening infections.

## Supporting Information

S1 Table Distribution of patients with proven/probable infections. (DOCX)
