# Supplementary material for: Rapid Recovery of CD3+CD8+ T Cells on Day 90 Predicts Superior Survival after Unmanipulated Haploidentical Blood and Marrow Transplantation
Source: PLoS One. 2016 Jun 8;11(6):e0156777. doi: 10.1371/journal.pone.0156777 (PMC4898737; doi:10.1371/journal.pone.0156777)
Supplement: S1 Table — (DOCX) [file pone.0156777.s001.docx]

**S1 Table. Distribution of patients with proven/probable infections.**

| Infection | CD3^+^CD8^+^-90≥375 cells/μL  (n=137) | CD3^+^CD8^+^-90<375 cells/μL  (n=77) |
| --- | --- | --- |
| Bacterial infections |  |  |
| Gram-negative |  |  |
| Escherichia coli | 5 | 8 |
| Pseudomonas aeruginosa | 0 | 2 |
| Klebsiella pneumoniae | 6 | 7 |
| Baumanii | 1 | 2 |
| Other | 1 | 3 |
|  |  |  |
| Gram-positive |  |  |
| Staphylococcus epidermidis | 2 | 3 |
| Staphylococcus aureus | 1 | 1 |
| Enterococcus faecalis | 2 | 3 |
| Enterococcus faecium | 0 | 1 |
| Other | 1 | 2 |
| Fungal infections |  |  |
| Aspergillus flavus | 4 | 5 |
| Aspergillus fumigatus | 3 | 2 |
| Mucor mycosis | 2 | 1 |
| Candida albicans | 6 | 8 |
| Other | 3 | 3 |
